# Prosthetic valve endocarditis caused by Pseudomonas aeruginosa with variable antibacterial resistance profiles: a diagnostic challenge

**DOI:** 10.1186/s12879-019-4164-3

**Published:** 2019-06-17

**Authors:** Nicolas Gürtler, Michael Osthoff, Florian Rueter, Daniel Wüthrich, Lukas Zimmerli, Adrian Egli, Stefano Bassetti

**Affiliations:** 1 Division of Internal Medicine and Department of Clinical Research, University Hospital Basel, University of Basel, Basel, Switzerland; 2 Department of Cardiac Surgery, University Hospital Basel, University of Basel, Basel, Switzerland; 3 Clinical Microbiology, University Hospital Basel, University of Basel, Basel, Switzerland; 4 Department of Internal Medicine, Kantonsspital Olten, Olten, Switzerland

**Keywords:** Endocarditis, *Pseudomonas aeruginosa*, Gram-negative bacilli, Transcatheter aortic valve implantation, Resistance profile, Case report

## Abstract

**Background:**

Infective endocarditis (IE) caused by gram-negative bacilli is rare. However, the incidence of this severe infection is rising because of the increasing number of persons at risk, such as patients with immunosuppression or with cardiac implantable devices and prosthetic valves. The diagnosis of IE is often difficult, particularly when microorganisms such as *Pseudomonas aeruginosa*, which rarely cause this infection, are involved. One of the mainstays for the diagnosis of IE are persistently positive blood cultures with the same bacteria, while polymicrobial bacteremia usually points to another cause, e.g. an abscess. The antimicrobial resistance profile of some *P. aeruginosa* strains may change, falsely suggesting an infection with several strains, thus further increasing the diagnostic difficulties.

**Case presentation:**

A 66-year old male patient who had a transcatheter aortic valve implantation (TAVI) one year previously developed fever seven days after an elective inguinal hernia repair. During the following four weeks, *P. aeruginosa* with different antibiotic resistance profiles was repeatedly isolated from blood cultures. Repeated trans-esophageal echocardiograms (TEE) were negative and an infection by different *P. aeruginosa* strains was suspected. Extensive diagnostic workup for an infectious focus was performed with no results. Finally, an oscillating mass on the aortic valve was detected by TEE five weeks after the initial positive blood cultures. *P. aeruginosa* endocarditis was confirmed by culture of the surgically removed valve. Whole genome sequencing of the last two *P. aeruginosa* isolates (valve and blood culture) revealed identical strains, with genome mutations for AmpR, AmpD and OprD.

**Conclusions:**

The diagnosis of prosthetic valve endocarditis is particularly difficult for several reasons. The modified Duke criteria have a lower sensitivity for patients with prosthetic valve endocarditis and the infection may be caused by “unusual” pathogens such as *P. aeruginosa*. Patients with repeatedly positive blood cultures should make clinicians suspicious for endocarditis even if imaging studies are negative and if isolated pathogens are “unusual”. Repeatedly positive blood cultures for *P. aeruginosa* should be considered as “persistent bacteremia” (suspicious for IE) even in the presence of different antibiotic susceptibility patterns, since *P. aeruginosa* might rapidly activate or deactivate resistance mechanisms depending on antibiotic exposition.

## Background

Infective endocarditis (IE) remains a serious disease that is still associated with significant morbidity and mortality, despite diagnostic and surgical advances [1–3]. Early stages of IE often lack distinct findings. The clinician’s ability to associate miscellaneous hints, including risk factors for acquisition of IE, is a key factor for rapid diagnosis. Fever, a new murmur or worsening of a known murmur and less often (< 5% of cases) cutaneous manifestations (Janeway lesions, Osler nodes) are typical clinical signs, but patients with IE mostly present either with an unspecific “sepsis syndrome” (in the case of acute IE) or with a subacute illness without typical signs [4]. New diagnostic tools such as cardiac computed tomography (CT) scanning or 18-fluorodeoxyglucose positron emission tomography (^18^FDG-PET)/CT are promising but expensive, and their precise role remains to be established [3]. The modified Duke criteria are of central importance for the evaluation of patients with suspected IE and include as major microbiological criterion “persistently positive blood cultures” [5].

*P. aeruginosa* is a very rare cause of endocarditis. In a recent Italian prospective cohort study (2004–2011) only 13 of 1722 IE episodes (0.75%) were caused by *Pseudomonas* species (including two co-infections) [6], compared with 11 of 2761 IE episodes (0.4%) in an international prospective cohort study analyzing IE cases from 2000 to 2005 [7]. However, the incidence of IE is increasing, in part because of more frequent use of cardiac implantable electronic devices and also because patients receiving transcatheter valve replacement may be at higher risk for IE [3, 4]. In the United States, the incidence of IE increased steadily from 11 to 15 per 100′000 population in the years 2000 to 2011, and the proportion of IE due to gram-negative bacteria increased from 5.3 to 8.2% [8]. Moreover, IE is currently health-care acquired in > 25% of cases [3].

For the same reasons, the epidemiology is changing also specifically for *P. aeruginosa* IE. Historically *P. aeruginosa* IE was associated with intravenous drug use [9]. However, a shift towards health care associated *P. aeruginosa* IE has been observed, in particular in patients with pacemaker or prosthetic valve implantation [2, 10]. Already the large cohort study by Morpeth et al. from 2000 to 2005 showed that most non-HACEK (species other than *Haemophilus* species, *Aggregatibacter actinomycetemcomitans*, *Eikenella corrodens*, and *Kingella* species) gram-negative bacilli IE were health-care associated (57%), while injection drug use was rare (4%) [7]. In addition, the above-mentioned more recent Italian cohort study confirmed that a genitourinary infection focus, immunosuppressive therapy, and an indwelling cardiac implantable electronic device, but not intravenous drug use, were associated with IE caused by non-HACEK gram-negative bacilli [6].

Treatment of *P. aeruginosa* IE is difficult and complicated by biofilm formation and by the possible emergence of antibiotic resistance during treatment because of genetic polymorphisms leading for example to the increased expression of cephalosporinases, changes in efflux pump regulators, or reduced porin expression [10, 11]. Therefore, combination antibiotic therapy is recommended and indication and timing of surgical treatment should be carefully assessed [3, 10].

## Case presentation

**Fig. 1:**
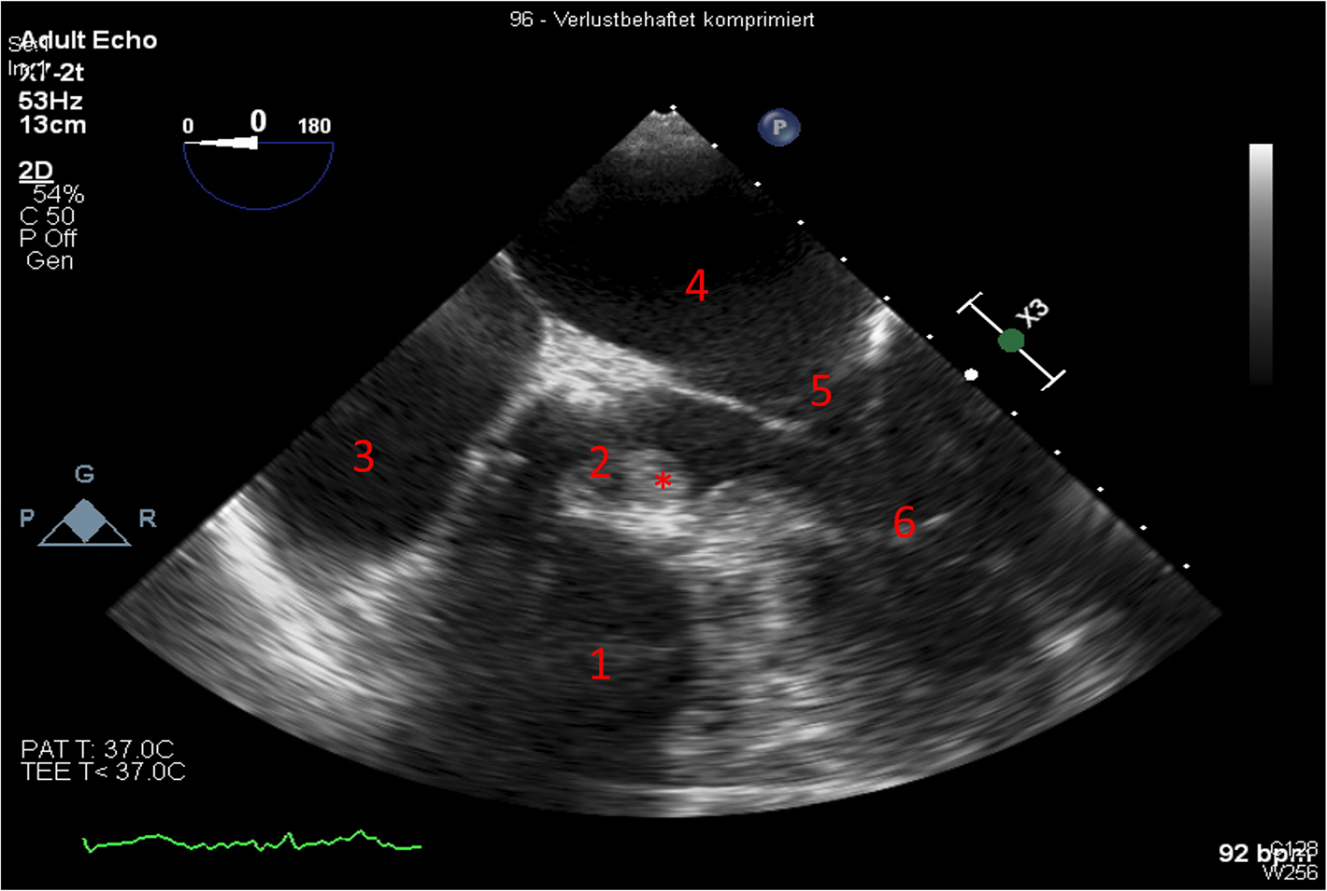
TEE at day 40 with four-chamber view. *free-floating mass attached to the aortic valve. ^1^right ventricle, ^2^aortic valve, ^3^right atrium, ^4^left atrium, ^5^mitral valve, ^6^left ventricle

**Fig. 2:**
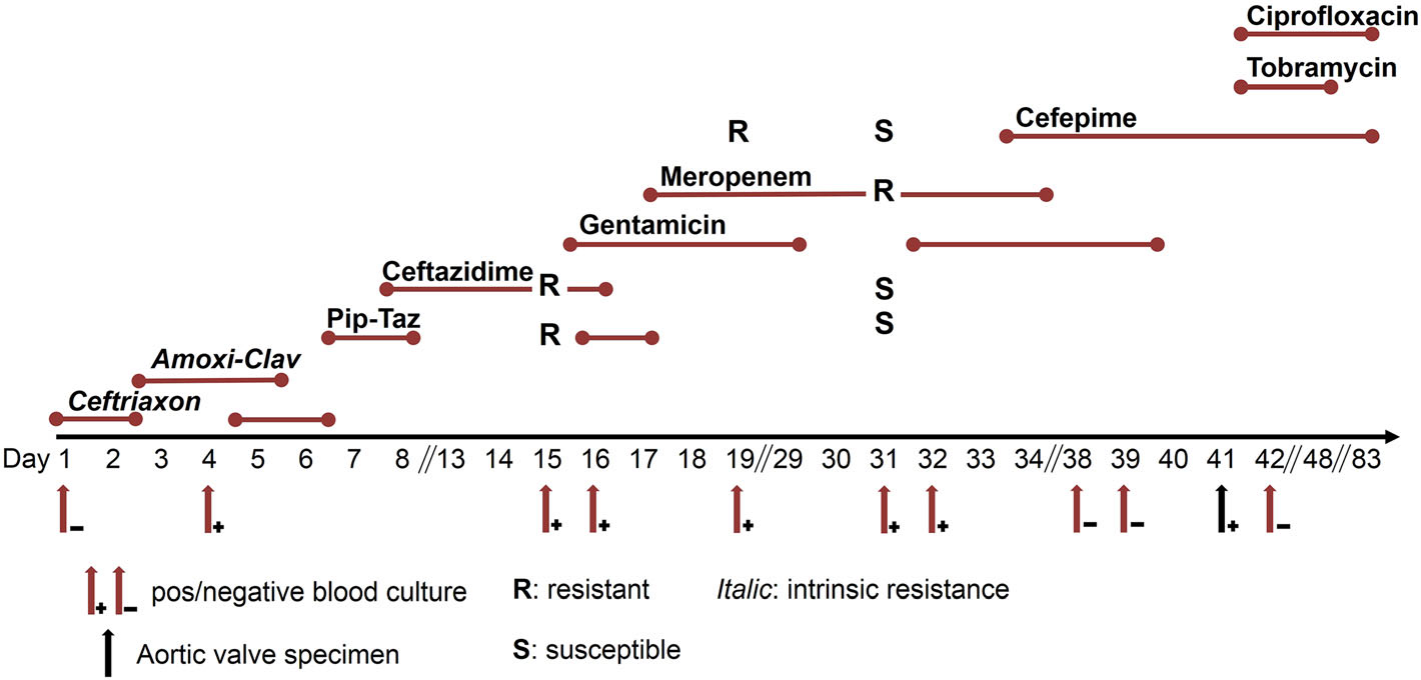
Timeline of hospital stay with display of blood culture drawings (red arrow), aortic valve specimen (black arrow), antibiotic agents with duration and resistance pattern (reading example: ceftazidime was tested resistant at day 15 and tested susceptible at day 31)

A 66-year old male patient presented to the emergency department with pain in the lower abdomen and a temperature of 38.6 °C. One week previously, after an incisional hernia repair, he had required a urinary catheter due to urinary retention. His past medical history was significant for a transcatheter aortic valve implantation (TAVI) due to a severe aortic stenosis 1 year earlier, and psoriasis vulgaris. On admission, the patient had a transurethral urinary catheter in place. The physical examination was normal, except for a febrile temperature and lower abdominal pain. The C-reactive protein was only mildly elevated to 16 mg/L (normal range < 10 mg/L), and mild pyuria (10–20 leucocytes per field of view) and hematuria (5–10 erythrocytes per field of view) were present. A catheter-associated urinary tract infection was suspected. Treatment with intravenous ceftriaxone (2 g qd) was initiated and changed after 3 days to intravenous amoxicillin-clavulanate (2.2 g tid). The patient continued to spike fevers up to 39.8 °C. Initial blood cultures were negative, but a repeated set of blood and urine cultures on day four was positive for *P. aeruginosa* (susceptible to all antibiotics tested, including piperacillin-tazobactam and ceftazidime). The antibiotic treatment was changed to intravenous piperacillin-tazobactam and later to ceftazidime. CT scans of the thorax and abdomen were unremarkable. A trans-esophageal-echocardiography (TEE) requested because of persistent fever, did not reveal any vegetation on the heart valves or other signs of infective endocarditis. Repeated blood cultures on day 15 were again positive for *P. aeruginosa*. However, now, susceptibility testing indicated resistance to piperacillin-tazobactam and ceftazidime. The treatment was changed accordingly to meropenem and gentamicin. Besides a mild fatigue, the patient had no localizing symptoms, and repeated TEE and abdominal and thoracic CT scans did not reveal any focus of infection. *P. aeruginosa* isolated from a blood culture on day 19 showed additional resistance to cefepime. On day 31, *P. aeruginosa* isolated from another blood culture changed its resistance profile one more time, now being again susceptible to piperacillin-tazobactam, ceftazidime and cefepime, but resistant to carbapenems. Antibiotic therapy was switched to cefepime and gentamicin. An ^18^FDG-PET/CT was not able to identify any focus of infection. After 5 weeks, the patient was transferred to a tertiary care university hospital. On day 40, a free-floating mass (12 × 8 mm) was identified on the aortic valve on TEE examination, and *P. aeruginosa* prosthetic valve endocarditis was diagnosed (Fig. 1). At this point, the patient was still febrile. Laboratory studies showed a leukocyte count of 8.9 × 10^9^/L (normal range 3.5–10.0 × 10^9^/L) and a C-reactive protein of 66.2 mg/L. The following day the patient successfully underwent surgical prosthetic valve replacement. The culture of the removed valve was positive for *P. aeruginosa*. Definitive antibiotic therapy consisted of intravenous cefepime, tobramycin and ciprofloxacin for additional 6 weeks (Fig. 2). The patient recovered quickly after the valve replacement and left our hospital for rehabilitation 9 days after surgery. He was doing well at the 3-month follow-up.

**Fig. 3:**
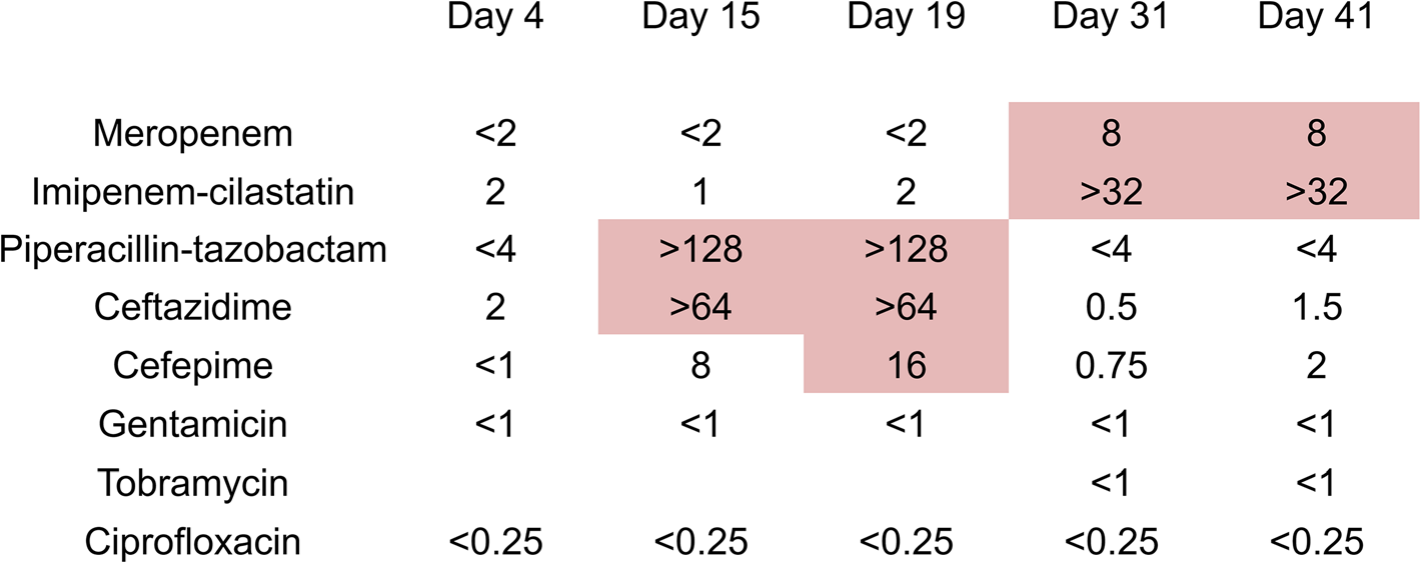
MICs of selected antibiotic agents, red markings for in vitro resistance

*P. aeruginosa* isolates from blood cultures at day 31 and from the culture of the removed valve at day 41 were analyzed using whole genome sequencing (MiSeq Illumina). No genetic differences could be detected between the two isolates (cgMLST showed zero allelic differences, both strains are ST 244), providing clear evidence that the *P. aeruginosa* isolates from the blood and the aortic valve were from the same strain. We could genotypically detect mutations in the following genes: transcriptional *AmpR* and beta-lactamase expression regulator *AmpD*, both linked to resistance against ceftazidime and piperacillin-tazobactam. In addition, we could also detect a mutation in *OprD*, which is linked to carbapenem resistance. This correlates with the phenotypic findings and is in line with findings in the literature. Unfortunately, the *P. aeruginosa* isolated from the first three positive blood cultures had already been discarded and were not available for further analysis (Fig. 3).

## Discussion and conclusions

We present a case of a prosthetic valve endocarditis with *P. aeruginosa*. This infection was difficult to diagnose and to treat. Initially, the presence of *P. aeruginosa* in the blood and urine after recent surgery with postoperative urinary retention suggested a surgical site infection or a urinary tract infection. *P. aeruginosa* accounts for 10% of healthcare-associated urinary tract infections and roughly 6% of surgical site infection in the USA [12]. Subsequently, IE was strongly suspected because of persistent bacteremia, the presence of a prosthetic valve, and lack of an alternative focus of infection (such as an abscess). Despite two TEEs and a ^18^FDG-PET/CT scan, it took more than a month until a final diagnosis was made. The initial isolation of *P. aeruginosa* from the urine and blood cultures (day 4) may be interpreted as catheter-associated urinary tract infection with bacteremia. Alternatively, as manifestation of endocarditis with persistent bacteremia and either secondary excretion of bacteria in the urine or concomitant urinary tract colonization. The empiric treatment with ceftriaxone may have contributed to the induction of resistance.

This case underlines the difficulty of diagnosing and treating IE caused by *P. aeruginosa*, a rare cause of IE and a pathogen able to form a biofilm and evade antimicrobial agents, but also able to develop resistance to multiple classes of antibiotics, even during the course of treatment [13, 14]. *P. aeruginosa* can become resistant to antibiotics by the acquisition of resistance genes on plasmids or through mutations under selection pressure that modify the expression and/or function of chromosomally encoded mechanisms [13]. Carmeli et al. observed that in around 10% of patients with *P. aeruginosa* infections, new resistances developed during antibiotic treatment, and identified imipenem as a main risk factor [15]. Strains with additional unclear resistance mechanisms (e.g. possible unstable de-repression of a chromosomal *AmpC* β-lactamase) leading to an unstable phenotype with changing antimicrobial resistance patterns have also been described [16]. Changing phenotypes may falsely suggest the presence of multiple strains, further hindering the diagnostic process. Molecular methods, such as whole-genome-sequencing (WGS) of isolated pathogens may be helpful to understand epidemiology (e.g. outbreaks), the course of the disease (e.g. differentiating relapse of an infection by the same strain from reinfection with different strains) and to identify the mechanisms of antibiotic resistance [11, 17]. In the presented case the development of resistance to imipenem and meropenem is most likely related to mutations of *OprD*, which mediates membrane porins, and/or to an activation/upregulation of efflux pumps [18]. The appearance and disappearance of resistance to piperacillin-tazobactam, ceftazidime and cefepime may be explained by a changing production of an inducible *AmpC* cephalosporinase (or an unstable de-repression of a chromosomal *AmpC* β-lactamase) [16]. As we were not able to analyze specimens of the first three positive blood cultures, it is also possible that different *P. aeruginosa* strains with variable genetic mutations were involved in the course of disease. A switch from multi-resistance to less resistance pattern in the same strain does not seem very likely.

Literature about unstable *P. aeruginosa* causing endocarditis is scarce. Lesho et al. described a similar case of unstable *P. aeruginosa*, in which resistance change was observed after storing *P. aeruginosa* ex vivo [16]. Contrary to our case, they observed only a one-time change of resistance pattern in the patient, whereas we described three changes over the course of the disease. However, our patient had a prolonged, persisting infection with changing selection pressures from antibiotics.

Domitrovic et al. described an infection by a *P. aeruginosa* with already broad resistance and gaining further resistance to third generation cephalosporins and piperacillin-tazobactam [11].

To our knowledge, this is the first case describing an infection with *P. aeruginosa*, which developed extensive cephalosporin, piperacillin-tazobactam and carbapenem resistance and partially lost this resistance. This highlights the ability of *P. aeruginosa* to switch on/off certain resistance mechanisms in shortest time.

A recent prospective cohort study of hospitalized patients with a cardiac device (including prosthetic heart valves) and a bacteremia showed that the risk of cardiac device-related infection is highest in patients with bacteremia due to *Staphylococcus aureus*, *P. aeruginosa* and *Serratia marcescens* [19]. In a patient with a cardiac device and bacteremia with *P. aeruginosa* one might therefore consider to start empiric therapy with a bactericidal combination of beta-lactams and aminoglycosides (preferably tobramycin), as recommended for *P. aeruginosa* endocarditis [20]. Although evidence for combination therapy in *P. aeruginosa* bacteremia is still lacking, combination therapy may also be necessary in absence of endocarditis in light of increasing resistance. Alternatively, optimized administration of beta-lactam antibiotics by continuous infusion coupled with therapeutic drug monitoring may be useful. New therapeutic approaches, as the combination of antibiotics and bacteriophages, might in the future improve the outcomes of treatment of IE caused by *P. aeruginosa* [21, 22].

In conclusion, the incidence of *P. aeruginosa* IE is increasing because of the growing number of persons at risk, such as patients with immunosuppression or with cardiac implantable devices and prosthetic valves. The diagnosis of this severe infection is often difficult and should be suspected, prompting adequate empiric antibiotic therapy, in all patients with persistent *P. aeruginosa* bacteremia, even if initial diagnostic tests are negative and particularly if implantable cardiac devices are present.

## Data Availability

The data used and analyzed in this case report is available from the corresponding author on reasonable request.

## Abbreviations

CT: Computed tomography
IE: Infective endocarditis
TAVI: Transcatheter aortic valve implantation
TEE: Trans-esophageal echocardiogram
